# Life problems in children and adolescents who self‐harm: findings from the multicentre study of self‐harm in England

**DOI:** 10.1111/camh.12544

**Published:** 2022-01-18

**Authors:** Ellen Townsend, Jennifer Ness, Keith Waters, Muzamal Rehman, Navneet Kapur, Caroline Clements, Galit Geulayov, Elizabeth Bale, Deborah Casey, Keith Hawton

**Affiliations:** ^1^ Self‐Harm Research Group School of Psychology University of Nottingham Nottingham UK; ^2^ Centre for Self‐harm and Suicide Prevention Research, Research and Development Centre Derbyshire Healthcare NHS Foundation Trust Kingsway Hospital Derby UK; ^3^ Centre for Mental Health and Safety Manchester Academic Health Sciences Centre University of Manchester Manchester UK; ^4^ Centre for Suicide Research University Department of Psychiatry University of Oxford and Oxford Health NHS Foundation Trust Warneford Hospital Oxford UK

**Keywords:** Life problems, self‐harm, children and adolescents, gender, repetition

## Abstract

**Background:**

Self‐harm, a significant and increasing global problem in children and adolescents, is often repeated and is associated with risk of future suicide. To identify potential interventions, we need to understand the life problems faced by children and adolescents, and by sub‐groups of younger people who self‐harm. Our aims were to include the following: (a) investigate the type and frequency of life problems in a large sample of children and adolescents who self‐harmed. (b) Examine whether problems differ between those who repeat self‐harm and those who do not.

**Methods:**

We analysed data for 2000 to 2013 (follow up until 2014) from the Multicentre Study of Self‐harm in England on individuals aged 11 to 18 years who presented to one of the five study hospitals following self‐harm and received a psychosocial assessment including questions about problems, which precipitated self‐harm.

**Results:**

In 5648 patients (12,261 self‐harm episodes), (75.5% female, mean age 16.1 years) the most frequently reported problems at first episode of self‐harm were family problems. Problems around study/employment/study and relationships with friends also featured prominently. The types of problems that precede self‐harm differed between late childhood/early adolescence. Abuse, mental health problems and legal problems significantly predicted repeat self‐harm for females.

**Conclusion:**

The most common problems reported by both genders were social/interpersonal in nature, indicating the need for relevant services embedded in the community (e.g. in schools/colleges). Self‐harm assessment and treatment choices for children and adolescents must take age and gender into account. To prevent future self‐harm, individualised supports and services are particularly needed for abuse, mental health and legal problems.

**Key Practitioner Message**

- Young people who self‐harm face a range of life problems.
- Detailed understanding of the nature of the problems faced by children and younger adolescents who self‐harm is limited.
- Family problems are significant for children and young people who self‐harm.
- Common life problems for young people who self‐harm are social or interpersonal in nature.
- Life problems vary by age, gender and whether self‐harm is repeated.
- Clinical supports and services for children and young people who self‐harm should be embedded in community settings.
- Life problems differ between children and younger adolescents compared with older adolescents, which should be accounted for in psychosocial assessments and recommended interventions.

## Introduction

Self‐harm (non‐fatal intentional self‐injury or self‐poisoning regardless of the intent of the act) (Hawton et al., 2003) is a common and increasing issue in young people in many countries (e.g. Cairns et al., 2019; Griffin et al., 2018; Morgan et al., 2017). Self‐harm is often repeated (Hawton, Bergen, & Kapur, 2012) and is the strongest risk factor for suicide (Hawton et al., 2020), which is the second highest cause of death globally in under 24‐year olds (Patton et al., 2009). Over 50% of children and adolescents who die by suicide have previously self‐harmed (Rodway et al., 2016). Understandably, self‐harm and suicide are major public health concerns targeted by global policy initiatives.

Most people who attend general hospitals for self‐harm face multiple life problems. Relationship problems have consistently been shown to be the most common issues preceding self‐harm (Liu & Miller, 2014). Younger people experience difficulties with family relationships and adults report problems with partners (Haw & Hawton, 2008; Townsend et al., 2016). Adolescents assessed in hospital most frequently report that problems preceding their self‐harm are relationship problems (with parents or boyfriend/girlfriend), and problems with school or work (Hawton, O’Grady, Osborn, & Cole, 1982). The US Youth Risk Behavior Survey showed that problems with alcohol were also associated with self‐reported suicide ideation and attempts (Baiden, Mengo, Boateng, & Small, 2019; Swahn & Bossarte, 2007).

Research on children and adolescents who self‐harm is needed to determine to what extent life problems differ according to key individual characteristics such as gender, age and whether self‐harm is repeated. Detailed research on problems leading to self‐harm in children and younger adolescents is lacking (Mitchell, Seah, Ting, Curtis, & Foster, 2018). Most questionnaire and interview‐based research on self‐harm has focused on older adolescents who can consent to research participation for themselves without parental consent (those over the age of 16 years in the UK). Here, we investigate life problems in a large sample of children and adolescents presenting to general hospital following self‐harm. We compare experiences of children and younger adolescents with older adolescents. Further, we have investigated whether specific life problems predict future self‐harm in young people. Understanding factors contributing to repeat self‐harm is vital in identifying effective interventions and in preventing suicide (Hawton et al., 2012, 2020). Contemporary models posit that life events are crucial in the ‘Premotivational Phase’ leading to self‐harmful behaviour (O’Connor & Kirtley, 2018).

The Multicentre Study of Self‐harm in England collects data across a range of life problems known to be important in leading to self‐harm. During the course of a comprehensive psychosocial assessment the life problems faced by patients are explored and recorded (Townsend et al., 2016). We used data from the Multicentre Study to examine life problems reported by children and adolescents who self‐harm.

The aims of the study were to:

1. Investigate the type and frequency of life problems reported (including by gender and age).
2. Examine whether problems differ between those who repeat self‐harm and those who do not.

## Methods

### Study design and participants

Participants were identified through the Multicentre Study of Self‐Harm in England (Hawton et al., 2007). The Multicentre Study is a prospective, collaborative study based in Oxford, Manchester, and Derby. Data are systematically collected on all presentations for self‐harm to the emergency departments in these cities. Data collection began in 2000 and is ongoing. We used data on individuals aged 11–18 years who presented with self‐harm to five general hospital emergency departments (EDs) in Oxford (1 hospital), Manchester (3) and Derby (1), between 1^st^ January 2000 and 31^st^ December 2013, with follow‐up to the end of 2014. Each centre has an established, robust monitoring system to collect data on all episodes of self‐harm presenting to the ED. The three centres use a standard definition of self‐harm, which is: “any act of intentional, non‐fatal, self‐poisoning or self‐injury, regardless of type of motivation or degree of suicidal intent” (Hawton et al., 2003). Given the overlap between self‐harm with and without suicidal intent, we do not distinguish suicide attempts from non‐suicidal self‐injury. Rather, we treat these behaviours as dimensional and existing on a continuum (Orlando, Broman‐Fulks, Whitlock, Curtin, & Michael, 2015).

### Life problems reported in psychosocial assessment

We included data on life problems for episodes where a full psychosocial assessment, a core element of standard care recommended in National Institute for Health and Care Excellence (NICE) guidance (Kendall, Taylor, Bhatti, Chan, & Kapur, 2011) was conducted by a mental health specialist. This is because reliable information on life problems was not available in the majority of cases where a psychosocial assessment was not conducted. In all three study centres, clinicians discuss life problems, which precipitated self‐harm with patients during the psychosocial assessment, which are then recorded by either clinicians themselves on a checklist (Oxford, Derby) or by researchers from the text arising from the assessment (Manchester).

The life problem categories used in the study were as follows: relationship with boyfriend/girlfriend; relationship with family; relationship with others; employment/ study; financial; housing; legal; physical health; mental health; bereavement; consequences of abuse (e.g. negative self‐esteem, difficulties establishing close relationships).

In two of the study centres (Oxford and Derby), there were also categories for problems with alcohol, drugs and “all other” problems (e.g. trauma experiences). Multiple life problems could be recorded at each episode, with each problem category coded as: yes, no, not known, or data missing. If every life problem category was recorded as not known or was missing for an episode, that episode was excluded from further analysis. The data are presented here are based on each patient’s first assessed episode between 1st January 2000 and 31st December 2013 (for cross‐sectional analyses), followed up until December 2014 (for longitudinal analyses).

### Age‐related differences

We assessed two age groups, 11–15 and 16–18 years, to reflect differing schooling (national exams taken at age 16 years—General Certificate of Secondary Education and 18 years—Advanced Level qualifications), developmental stages (childhood‐early adolescence vs. middle‐late adolescence) and legal status in the United Kingdom with regard to sexual activity (age of legal consent is 16 years), drinking alcohol (legal drinking age is 18 years) and ability to consent to research (over 16 years in the UK).

### Repetition of self‐harm in young people

Repetition of self‐harm was defined as any further episode of self‐harm by an individual resulting in presentation to the same study hospitals (in Manchester, this could be any of the three hospitals) within 12 months of their first assessed episode, the period when most episodes of repetition occur (Townsend et al., 2016). Included in these analyses were individuals who presented to the hospitals between January 2000 and December 2013 for the first time and followed up until 31^st^ December 2014, which ensured that all patients were followed up for at least 12 months.

### Ethical approval

The self‐harm monitoring systems in Oxford and Derby have Health Research Authority and NHS Research Ethics Committee approvals to collect data for both local and multicentre projects. In Manchester, the monitoring is conducted as part of a clinical audit system, ratified by the local research ethics committee. All centres have approval under section 251 of the NHS Act (2006) to collect patient‐identifiable data without patient consent.

### Statistical analyses

To investigate the type and frequency of life problems reported (including by gender and age), cross‐sectional categorical data on problems were analysed at an individual level with χ^2^. Cramer’s V post hoc tests were used to measure effect size (strength of any association). Where there were more than two categories, analysis of standardised residuals identified where more or fewer patients than expected by chance reported having a particular problem (positive or negative residuals >1.96 respectively). We corrected for multiple comparisons for each analysis using the Bonferroni method. *T*‐tests were used to test for the differences in number of life problems reported between genders. To examine whether problems differ between those who repeat self‐harm and those who do not, Logistic Regression was used (no repeat episode vs. repeat episode), within twelve months of each first assessed episode (longitudinal data). We also examined repetition using χ^2^ analysis to compare life problems for those who had not repeated, those with 1 to 2 further episodes and those with 3 or more episodes. Missing data were excluded from the relevant parts of the analysis. All analyses were conducted in SPSS v.24.

## Results

Life problem data were not available for 3644/9284 (39%) individuals in the study period because they did not receive a psychosocial assessment (and hence were excluded from the study). The included and excluded samples were similar in terms of age at their first assessed episode (excluded: mean 16.0 years *SD* 1.7 vs. included mean = 16.1 years *SD* 1.6; *t* = 1.01, *p* = .31) at their first assessed episode. However, a somewhat greater proportion of females were in the included sample (75.5% vs. 72.8% in the excluded individuals χ^2^ = 8.86, *p* < .01). A greater proportion of those not receiving an assessment presented with self‐injury (usually self‐cutting) as the method of harm at their first assessed episode (self‐poisoning excluded 73.2% vs. included 80.5%; self‐injury excluded 23.8% vs. included 13.2%; both self‐poisoning and self‐injury excluded 3.0% vs. included 6.3% (χ^2^ = 189.91, 2 df, *p* < .001)).

### Self‐harm summary data

During the 14‐year initial study period (2000–2013), 9284 individuals aged 11 to 18 years attended the EDs, with a total of 17,740 episodes of self‐harm between 2000 and 2014 (including the extra 12 months of follow up). Of these patients, 61.0% had at least one episode assessed by a mental health specialist, giving a final study sample of 5648 patients (with 12,261 episodes between 2000 and 2014). At their first assessed episode within the study period, 2441 (54.4%) individuals reported having previously self‐harmed.

A large majority (75.5%) of those assessed were female, with gender unknown for three individuals. Over two‐thirds of the sample were aged 15 to 18 years (3796, 67.2%), with the mean age at first assessed episode being 16.1 years (*SD* 1.6).

Over three quarters (80.5%, *n* = 4545) of first assessed episodes involved self‐poisoning alone, 13.2% (*n* = 747) self‐injury (most commonly self‐cutting) and 6.3% (*n* = 356) both self‐poisoning and self‐injury. In terms of ethnicity, the majority of the sample were white (70.8%, *n* = 3997), 8.5% (*n* = 484) were from an ethnic minority group and the ethnicity was unknown for 20.7% (*n* = 1167). A third of patients (29.2%. *n* = 1648) were identified as having a single life problem preceding self‐harm, whereas 54.9% (*n* = 3100) had multiple problems. Life problem data were missing for 15.9% (*n* = 900) of patients and so these cases were excluded from further analyses. Males and females had similar mean numbers of problems (males: mean = 2.3; females: mean = 2.2, *t* = 0.96, *p* = .34).

### Frequency of life problems by gender

For both genders, problems with family were the most common life problem (see Table 1) but were more prevalent in females than males in the older age group. Compared with males, females in both age groups were more likely to have problems with relationships with friends and older females were more likely to report problems with their families. Males of both age groups were more likely than females to have problems relating to alcohol, housing and finances. Older males were more likely than females to have legal or drug‐related problems, whereas older females were more likely than males to report problems relating to abuse (Table 1).

**Table 1:** Gender comparison of life problem prevalence at first assessed episode for 11 to 15 and 16‐ to 18‐year olds

Life Problem: 11‐ to 15‐year olds	Males (*N* = 208, 16.3%) *N* (%)	Females (*N* = 1067, 83.7%) *N* (%)	Total (*N* = 1275) *N* (%)	χ^2^ (Cramer’s v)
Relationship with family members	128 (61.5)	688 (64.5)	816 (64.0)	0.65 (0.02)
Relationship with friends/others	40 (19.2)	369 (34.6)	409 (32.1)	18.83* (0.12)
Employment/study	71 (34.1)	306 (28.7)	377 (29.6)	2.49 (0.04)
Relationship with boy/girlfriend	39 (18.8)	153 (14.3)	192 (15.1)	2.65 (0.05)
Consequences of abuse	28 (13.5)	136 (12.7)	164 (12.9)	0.08 (0.01)
Mental health	29 (13.9)	124 (11.6)	153 (12.0)	0.89 (0.03)
Bereavement	11 (5.3)	75 (7.0)	86 (6.7)	0.84 (0,03)
Housing	19 (9.1)	48 (4.5)	67 (5.3)	7.51* (0.08)
Physical health	5 (2.4)	44 (4.1)	49 (3.8)	1.39 (0.03)
Financial	8 (3.8)	15 (1.2)	23 (1.8)	5.85* (0.07)
Legal	5 (2.4)	16 (1.5)	21 (1.6)	0.88 (0.03)

Oxford & Derby only	*N* = 147, 14.6%	*N* = 859, 85.4%	*N* = 1006	
All other	29 (19.7)	162 (18.9)	191 (19.0)	0.62 (0.01)
Alcohol	21 (14.3)	50 (5.8)	71 (7.1)	13.71* (0.12)
Drugs	9 (6.1)	43 (5.0)	52 (5.2)	0.32 (0.02)

Life problem: 16‐ to 18‐year olds	Males (*N* = 982, 28.3%) *N* (%)	Females (*N* = 2490, 71.7%) *N* (%)	Total (*N* = 3472) *N* (%)	χ^2^ (Cramer’s V)
Relationship with family members	418 (42.6)	1212 (48.7)	1630 (46.9)	10.55* (0.16)
Relationship with boy/girlfriend	369 (37.6)	978 (39.3)	1347 (38.8)	0.86 (0.02)
Employment/study	258 (26.4)	618 (24.8)	877 (25.3)	0.90 (0.02)
Mental health	168 (17.1)	406 (16.3)	574 (16.5)	0.33 (0.01)
Relationship with friends/others	133 (13.5)	410 (16.5)	543 (15.6)	4.56*(0.04)
Consequences of abuse	80 (8.1)	320 (12.9)	400 (11.5)	15.29* (0.07)
Housing	129 (13.1)	263 (10.6)	392 (11.3)	4.66* (0.04)
Bereavement	78 (7.9)	209 (8.4)	287 (8.3)	0.19 (0.01)
Financial	96 (9.8)	178 (7.1)	274 (7.9)	6.69* (0.05)
Legal	61 (6.2)	47 (1.9)	108 (3.1)	43.70* (0.11)
Physical health	41 (4.2)	129 (5.2)	170 (4.9)	1.53 (0.02)

Oxford & Derby only	*N* = 536, 27.1%	*N* = 1441, 72.9%	*N* = 1977	
All other	105 (19.6)	309 (21.4)	414 (20.9)	0.81 (0.02)
Alcohol	114 (21.3)	183 (12.7)	297 (15.0)	22.47* (0.11)
Drugs	86 (16.0)	92 (6.4)	178 (9.0)	44.50* (0.15)

*Statistically significant results after Bonferroni correction of *p* < .002 (there are 28 comparisons, so correction applied is 0.05/28 = 0.002).

### Frequency of life problems by age group

Problems with families, relationship with friends/others, studying, and consequences of abuse were more prevalent in younger than older males. In the older age group, relationships with boy/girlfriends, financial, legal problems and drug problems were more common (Table 2). In females, there was a similar pattern, with problems with families, friends and studying being more common in 11‐ to 15‐year olds and problems concerning boy/girlfriends being more common in 16‐ to 18‐year olds, along with, housing and financial problems (Table 2).

**Table 2:** Age group comparisons for life problems at the time of first assessed episode by gender

Life problem: males	11‐ to 15‐year olds *N* = 208, 17.5% *N* (%)	16‐ to 18‐year olds *N* = 982, 82.5% *N* (%)	χ^2^ (Cramer’s V)
Relationship with family members	128 (61.5)	418 (42.6)	24.88* (0.15)
Employment/study	71 (34.1)	259 (26.4)	5.16* (0.07)
Relationship with friends/others	40 (19.2)	133 (13.5)	4.47* (0.06)
Relationship with boy/girlfriend	39 (18.8)	369 (37.6)	27.00* (0.15)
Mental health	29 (13.9)	168 (17.1)	1.26 (0.03)
Consequences of abuse	28 (13.5)	80 (8.1)	5.88* (0.07)
Housing	19 (9.1)	129 (13.1)	2.52 (0.05)
Bereavement	11 (5.3)	78 (7.9)	1.75 (0.04)
Financial	8 (3.8)	96 (9.8)	7.57* (0.08)
Physical health	5 (2.4)	41 (4.2)	1.45 (0.04)
Legal	5 (2.4)	61 (6.2)	4.75* (0.06)

Oxford & Derby only	*N* = 147, 21.5%	*N* = 536, 78.5%	
Other	29 (19.7)	105 (19.6)	0.01 (0.01)
Alcohol problem	21 (14.3)	114 (21.3)	3.55 (0.07)
Drug problem	9 (6.1)	86 (16.0)	9.49* (0.12)

Life problem: females	11‐ to 15‐year olds *N* = 1067, 30% *N* (%)	16‐ to 18‐year olds *N* = 2490, 70% *N* (%)	χ^2^ (Cramer’s V)
Relationship with family members	688 (64.5)	1212 (48.7)	74.98* (0.15)
Relationship with friends/others	369 (34.6)	410 (16.5)	143.341* (0.20)
Employment/study	306 (28.7)	618 (24.8)	5.79* (0.04)
Relationship with boy/girlfriend	153 (14.3)	978 (39.3)	214.20* (0.25)
Consequences of abuse	136 (12.7)	320 (12.9)	0.01(0.01)
Mental health	124 (11.6)	406 (16.3)	12.92*(0.06)
Bereavement	75 (7.0)	209 (8.4)	1.89 (0.02)
Housing	48 (4.5)	263 (10.6)	34.42*(0.10)
Physical health	44 (4.1)	129 (5.2)	1.80 (0.02)
Legal	16 (1.5)	47 (1.9)	0.66 (0.01)
Financial	15 (1.4)	178 (7.1)	48.00* (0.12)

Oxford & Derby only	*N* = 859, 37.4%	*N* = 1441, 62.6%	
Other	162 (18.9)	309 (21.4)	2.21 (0.03)
Alcohol problem	50 (5.8)	183 (12.7)	27.97* (0.11)
Drug problem	43 (5.0)	92 (6.4)	1.85 (0.03)

*Statistically significant results after Bonferroni correction of *p* < .002 (there are 28 comparisons, so correction applied is 0.05/28 = 0.002).

### Life problems and repetition of self‐harm

Overall, 1058 (18.7%) (one in five) of the included individuals had a repeat presentation to the original study hospital within 12 months of their first assessed episode in the study period. This included 660 (11.7%) with one repeat presentation, 298 (4.3%) with two or three repeat episodes and 100 (2.7%) who repeated four or more times.

Repetition of self‐harm in females was associated with individuals having more life problems (mean 2.38 vs. 2.21, *t* = 2.94, 1 *df*, *p* < .01). The difference was not significant for males (2.43 vs. 2.25, *t* = 1.77, 1 *df*, *p* = .08). Females who repeated self‐harm were more likely than those who did not to have problems with consequences of abuse, mental health and legal problems. This was also the case for “all other problems” in Oxford and Derby. Problems with study/employment and relationships with a boy/girlfriend were more frequent in females who did not repeat self‐harm than in those who did repeat. (Table 3). Males who repeated self‐harm were more likely than those who did not to have problems with consequences of abuse and housing. This was also true for drug‐related problems in Oxford and Derby (Table 3).

**Table 3:** Prevalence of life problems at the time of first assessed episode by repetition status

Life problem: males	Repetition status (within 12 months)			χ^2^ (Cramer’s V)
	No repeat presentation *N* = 967, 81.3% *N* (%)	1 to 2 repetitions *N* = 193, 16.2% *N* (%)	3 or more repetitions *N* = 30, 2.5% *N* (%)	
Relationship with family members	448 (46.3)	82 (42.5)	16 (53.3)	χ^2^ = 1.64 (0.04)
Relationship with friends/others	143 (14.8)	30 (15.5)	0 (0)	χ^2^ = 5.31 (0.07)
Employment/study	279 (28.9)	45 (23.3)	6 (20.0)	χ^2^ = 3.38 (0.05)
Relationship with boy/girlfriend	341 (35.3)	61 (31.6)	6 (20.0)	χ^2^ = 3.74 (0.06)
Consequences of abuse	74 (7.7)‐‐‐	28 (14.5)++	6 (20.0)+++	χ^2^ = 13.61* (0.11)
Mental health	153 (15.8)	36 (18.7)	8 (26.7)	χ^2^ = 3.21 (0.05)
Bereavement	72 (7.4)	15 (7.8)	2 (6.7)	χ^2^ = 0.05 (0.01)
Housing	105 (10.9)‐‐‐	32 (16.6)	11 (36.7)+++	χ^2^ = 21.43* (0.13)
Physical health	36 (3.7)	8 (4.1)	2 (6.7)	χ^2^ = 0.72 (0.03)
Legal	46 (4.8)	17 (8.8)	3 (10.0)	χ^2^ = 6.21 (0.07)
Financial	83 (8.6)	20 (10.4)	1 (3.3)	χ^2^ = 1.77 (0.04)

Oxford & Derby only	*N* = 557, 81.6%	*N* = 110, 16.1%	*N* = 16, 2.3%	
Other	109 (19.6)	23 (20.9)	2 (12.5)	χ^2^ = 0.63 (0.03)
Alcohol problem	105 (18.9)	26 (23.6)	4 (25.0)	χ^2^ = 1.61 (0.05)
Drug Problem	72 (12.9)	17 (15.5)	6 (37.5)++	χ^2^ = 8.11*(0.11)

Life problem: females	Repetition status (within 12 months)			χ^2^ (Cramer’s V)
	No repeat presentations *N* = 2888, 81.2% *N* (%)	1 to 2 repetitions *N* = 545, 15.3% *N* (%)	3 or more repetitions *N* = 124, 3.5% *N* (%)	
Relationship with family members	1530 (53.0)	307 (56.3)	63 (50.8)	χ^2^ = 2.42 (0.03)
Relationship with friends/others	636 (22.0)	109 (20.0)	34 (27.4)	χ^2^ = 3.38 (0.08)
Employment/study	778 (26.9)++	122 (22.4)‐	24 (19.4)	χ^2^ = 7.87* (0.05)
Relationship with boy/girlfriend	960 (33.2)+++	148 (27.2)‐	23 (18.5)‐‐	χ^2^ = 18.23* (0.07)
Consequences of abuse	326 (11.3)‐‐‐	105 (19.3)+++	25 (20.2)++	χ^2^ = 32.31*(0.10)
Mental health	373 (12.9)‐‐‐	121 (22.2)+++	36 (29.0)+++	χ^2^ = 51.42*(0.12)
Bereavement	236 (8.2)	42 (7.7)	6 (4.8)	χ^2^ = 1.87 (0.02)
Housing	239 (8.3)	59 (10.8)	13 (10.5)	χ^2^ = 4.22 (0.03)
Physical health	147 (5.1)	24 (4.4)	2 (1.6)	χ^2^ = 3.40 (0.03)
Legal	40 (1.4)‐‐‐	19 (3.5)+++	4 (3.2)++	χ^2^ = 13.20* (0.06)
Financial	162 (5.6)	26 (4.8)	5 (4.0)	χ^2^ = 1.12 (0.02)

Oxford & Derby only	*N* = 1869, 81.3%	*N* = 346, 15.0%	*N* = 85, 3.7%	
All other problems	366 (19.6)‐	77 (22.3)	28 (32.9)++	χ^2^ = 9.70*(0.07)
Alcohol problem	185 (9.9)	34 (9.8)	14 (16.5)	χ^2^ = 3.90 (0.04)
Drug problem	104 (4.5)	25 (7.2)	6 (7.1)	χ^2^ = 1.68 (0.03)

‐/+ Adjusted Standardised Residual score of >= 1.96 (*p* < .05); ‐‐/++ standardised residual score of >= 2.58 (*p* < .01); ‐‐‐/+++standardised residual score of >= 3.29 (*p* < .001).

*Statistically significant after Bonferroni correction.

Some problems associated with repetition in the above analyses might overlap with other problems. We therefore investigated whether certain problems were independently associated with repeated self‐harm during the 12 months following first presentations in the study period, using logistic regression analyses. Two separate regressions for males and females were conducted on problems shown to be significantly associated with repetition in Table 3. Models were adjusted for age and all other problems in the model (see Table 4). These analyses showed that having mental health problems, dealing with the consequences of abuse, facing legal issues and having ‘other problems’ were significantly associated with repetition of self‐harm in females. For males, there was a trend toward (dealing with the consequences of) abuse predicting repeated self‐harm, but this was not statistically significant.

**Table 4:** Multivariate Logistic Regression analyses of the independent associations between repetition of self‐harm within 12 months and life problems in 11‐ to 18‐year olds

Life problems males	Odds ratio	Confidence Interval (95%)		*p* value
		Lower	Upper	
Age	0.98	0.85	1.12	.76
Housing problems	1.53	0.89	2.64	.13
Legal problems	1.68	0.83	3.39	.15
Drug problems	1.35	0.80	2.30	.27
Consequences of abuse	1.67	0.92	3.01	.09
Constant	1.34	—	—	.82

Life problems females	Odds Ratio	Confidence interval (95%)		*p* value
		Lower	Upper	
Age	1.13	1.06	1.21	.00
Consequences of abuse	1.76	1.32	2.33	.00*
Housing problems	1.16	0.79	1.71	.45
Drug problems	1.18	0.76	1.82	.47
Employment/study problems	0.78	0.61	1.01	.06
Relationship with boy/girlfriend	0.89	0.69	1.16	.39
Mental health problems	2.39	1.82	3.14	.00*
Legal problems	3.35	1.67	6.72	.01*
Other problems	1.40	1.08	1.80	.01*
Constant	0.04	—	—	.00

* No repetition was the comparison category of the dependent variable. Models were adjusted for age.

## Discussion

This study demonstrates that life problems faced by children and younger adolescents preceding self‐harm differ by age and gender, and also between those who repeat self‐harm and those who do not. This has important implications for the assessment of children and adolescents who present to hospital for self‐harm and for provision of interventions.

The most common life problem reported was with family members. We have previously shown this for an older group of young people (Townsend et al., 2016) and research from the US indicates that problematic parenting (harsh punishment, poor attachment and low parental monitoring) predicts the onset of non‐suicidal self‐injury in adolescent females (Victor, Hipwell, Stepp, & Scott, 2019). Our findings highlight the need to help younger children and adolescents cope with family difficulties by equipping them with the skills/problem solving strategies to manage relationships and deal with difficult emotions. An obvious intervention would be family therapy, but while this approach does not help prevent further repetition of self‐harm (Cottrell, Wright‐Hughes, & Collinson, 2018; Harrington et al., 1998) it may have a positive impact on emotional symptoms, conduct problems, hyperactivity/inattention and prosocial behaviour (Cottrell et al., 2018), and family involvement is an important element of other psychosocial interventions (Kothgassner, Robinson, Goreis, Ougrin, & Plener, 2020). Qualitative research has shown that family and parental responses to a young person’s distress and self‐harming are very important (Ferrey et al., 2016) and a key area for future research (Wadman et al., 2018). A follow‐up study of a Youth‐Nominated Support Team Intervention with adolescents who attempted suicide demonstrated significantly reduced risk of future suicide in those who were supported by a caring adult mentor (using a problem‐solving approach) following their attempts (King et al., 2019).

### Life problems by age and gender

Our findings indicate that the types of problems preceding self‐harm differ in late childhood/early adolescence compared with later adolescence. The most common problems for both genders were those involving relationships, for example, with family, friends and girl/boyfriends. However, some interesting differences emerged when examining differences by age and gender. The younger age group females were significantly more likely to report problems with friends/others compared with males and also older females. This suggests that younger females especially require support with dealing with problematic peer relationships, which may be characterised by bullying or cyberbullying, both of which are associated with suicidal behaviour (Rodway et al., 2016). These findings are of particular significance since many young people who are struggling with self‐harm seek help from friends, indicating the need for peer‐group interventions (Gillies, Christou, & Dixon, 2018).

In the older age group, females were more likely than males to report problems dealing with the consequences of abuse and family problems, whereas males were more likely to report legal, financial, housing, drug and alcohol problems. Older males and females were more likely to report problems with boyfriends/girlfriends compared with younger males and females, whereas the younger age groups experienced more problems with their families. The findings of this study indicate the need for clinicians conducting psychosocial assessments and organising therapeutic interventions following self‐harm in children and adolescents to take age and gender into account. There is evidence that conducting ‘therapeutic assessments’ with young people, which includes a focus on key problems facing individuals, can increase treatment adherence (Ougrin et al., 2011).

Our findings also suggest that interventions are needed in the community where interpersonal problems (e.g. with friends) occur. School‐based Youth Awareness of Mental Health programmes are beneficial in preventing suicidal ideation and behaviour (Wasserman et al., 2015), although these have not yet been tested in the United Kingdom. In the United States, suicide prevention programs (e.g. Signs of Suicide) have been effective in reducing suicide attempts in school‐aged children (Schilling, Aseltine, & James, 2016). The ‘Mental Health Support Teams’ proposed in the NHS Long‐Term Plan would be ideally situated to support children and adolescents in schools.

### Life problems and Repetition of self‐harm

Repetition of self‐harm was common in this study. Legal problems and problems relating to the consequences of abuse were associated with increased risk of future self‐harm for both males and females. Specific types of problems were more predictive of future self‐harm than others in females but not in males. In particular, dealing with the consequences of abuse, having mental health problems, legal issues and a range of heterogeneous ‘other problems’ (e.g. miscarriage, facial scarring, low self‐esteem, etc.) conferred an increased risk of females repeating self‐harm. Dealing with the consequences of abuse and mental health problems may relate to long‐term distress and difficulties, which have significant implications for the type of therapeutic interventions that may be helpful. Treatments with extended contact periods, such as Mentalisation‐Based Therapy (MBT) and Dialectical Behavior Therapy (DBT), appear to be beneficial and could help those who repeat self‐harm (Witt et al., 2021). Given the preponderance of relationship issues reported in this study and in others, it is likely that Interpersonal Therapy (Walker, Shaw, & Turpin, 2018) could be helpful for young people who self‐harm, although to our knowledge, this has not yet been evaluated in an RCT with children and adolescents where repeated self‐harm is the main outcome measure (Witt et al., 2021).

### Limitations

Our sample was limited to those who attended hospital for self‐harm and received a psychosocial assessment. However, community‐based studies of adolescents who self‐harm also indicate that interpersonal problems are important contributory factors in this population (Tang et al., 2016).

A considerable number of young people in this study (39%) did not receive a psychosocial assessment following hospital attendance for self‐harm. Unfortunately, despite NICE guidance, which recommends that all patients should receive an assessment, many still do not, for a variety of reasons (Quinlivan et al., 2021). It is vital that all young people attending hospital for self‐harm receive a psychosocial assessment, not least because receiving an assessment can be associated with reduced repetition of self‐harm (Kapur et al., 2013).

The effect sizes for some of the differences detected in this study (e.g., between age groups and gender) were mostly relatively small despite conservative (Bonferroni corrected) tests of significance. We also did not have information about the duration or intensity of the life problems the young people were facing. Studies using Ecological Momentary Assessment methods would be useful to explore this issue.

Self‐harm is multidimensional and complex, we have not been able to investigate all relevant risk factors for young people; for example, the influence of social media (Marchant, Hawton, & Stewart, 2017) and victimization (Baldwin et al., 2019; Williams et al., 2021) will be important to investigate moving forward. Another important direction for future research would be to examine whether the ‘life problems’ experienced by young people who self‐harm are distinct from those experienced by those in other clinical samples (eg depressed children and adolescents), as this may have important implications for the design and implementation of interventions, although one might expect some degree of overlap given potential co‐morbidity.

Considerable strengths of the study were the size of the sample, the fact that the patients were identified in a consistent fashion over a lengthy time period, and that they were from a socioeconomically diverse population. However, we will have missed some young people who repeated self‐harm if they attended a hospital outside of the study areas or if they did not present to hospital following self‐harm (Geulayov et al., 2018).

## Conclusion

### Clinical implications

For children and adolescents, self‐harm usually occurs in the context of one or more life problems. It is notable that most common life problems found were largely social/interpersonal in nature: this has important implications for clinical services and suggests that very close links between health and other support services working with children and adolescents are required. Ideally services and supports should be integrated with multiagency working, including schools and colleges, and clear care pathways offering evidence‐based psychosocial, which include supports and services provided in schools and colleges. It is vital that all of those who work with children and adolescents who self‐harm see beyond life problems experienced and respond to the suffering, strong negative emotions and mental pain that these problems elicit. Compassionate responses that acknowledge and hold the distress, which underpins self‐harm are vital (Townsend, 2019).

## Ethical information

The self‐harm monitoring systems in Oxford and Derby have Health Research Authority and NHS Research Ethics Committee approvals to collect data for both local and multicentre projects. In Manchester, the monitoring is conducted as part of a clinical audit system, ratified by the local research ethics committee. All centres have approval under section 251 of the NHS Act (2006) to collect patient‐identifiable data without patient consent.
